# Control of Friction Laws in Tangential Adhesive Contacts by Surface Geometry

**DOI:** 10.3390/ma19081549

**Published:** 2026-04-13

**Authors:** Josefine Fritsch-Wilhayn, Khudoyar Buranov, Qiang Li, Ken Nakano, Valentin L. Popov

**Affiliations:** 1 Department of System Dynamics and Friction Physics, Technische Universität Berlin, 10623 Berlin, Germany; qiang.li@tu-berlin.de; 2 Department of Theoretical and Applied Mechanics, Samarkand State University, Samarkand 140104, Uzbekistan; 3 Faculty of Environment and Information Sciences, Yokohama National University, 79-7, Yokohama 240-8501, Japan; nakano@ynu.ac.jp

**Keywords:** friction, viscoelastic contact, adhesion, indenter geometry, tangential contact, Boundary Element Method

## Abstract

Adhesive quasi-static tangential contact between a rigid indenter and a linearly viscoelastic half-space is investigated numerically using the Boundary Element Method. The indenter geometry is described by a power-law profile including parabolic (*n* = 2), conical (*n* = 1), and sharp-tip (*n* = 1/2) indenters. Adhesion is incorporated through a stress-based detachment criterion with effective works of adhesion derived from an energetic approach for quasi-static viscoelastic contacts. During sliding, elements at the leading edge of the contact attach, while those at the trailing edge detach. Due to the viscoelastic response of the material, adhesion at the leading edge is weak, whereas adhesion at the trailing edge is significantly stronger. This asymmetry generates a tangential force acting at the contact boundary. Numerical simulations performed for different ratios of the shear moduli *G*_0_/*G*_1_ show that the friction force strongly depends on the indenter geometry and follows different power-law relations to the normal force: a one-third power for parabolic indenters, a square-root dependence for conical indenters, and a two-thirds power for sharp-tip indenters.

## 1. Introduction

The classical Amontons–Coulomb law of friction states that the friction force is proportional to the normal load and is independent of the apparent contact area and surface geometry [1,2]. While this empirical law successfully describes many macroscopic frictional systems, numerous studies have demonstrated its violation in many situations, for example, in contacts at small scales or involving soft materials [2,3]. In such cases, adhesive interactions can play a dominant role and strongly influence the frictional behavior [4,5].

Adhesion is frequently observed in contacts involving soft or viscoelastic materials [6,7]. In indentation experiments with viscoelastic materials, a characteristic adhesion hysteresis between the indentation and pull-off phases is often observed [8,9,10]. During indentation, the contact often appears very similar to a non-adhesive Hertzian contact, whereas during pull-off strong adhesive forces become evident. This behavior can be explained by the concept of the effective work of adhesion [11]. When an element enters into contact during indentation, the process corresponds to crack closing, for which the effective work of adhesion is very small. As a result, adhesion effects are largely suppressed and the contact behaves almost like a Hertzian contact. In contrast, during pull-off elements detach from the surface, which corresponds to crack opening. In this case the effective work of adhesion is much larger, leading to pronounced adhesive forces. This asymmetry between attachment and detachment is a fundamental feature of quasi-static adhesive contacts in viscoelastic materials.

A theoretical framework describing this phenomenon was recently developed in the form of an energetic detachment criterion for viscoelastic adhesive contacts [12,13]. The theory is based on the separation of time scales between rapid detachment and the slower viscous relaxation of the material. As a consequence, the instantaneous response during detachment is governed by the glass modulus of the material, which allows a Griffith-type energy balance to be applied even in dissipative media.

The same physical mechanism also applies to tangential sliding contacts [14]. During sliding, elements at the leading edge of the contact enter into contact with the surface, which is analogous to crack closing. At the trailing edge, elements detach from the surface, corresponding to crack opening. Because the effective works of adhesion associated with these two processes are very different in viscoelastic materials, the contact boundary becomes strongly asymmetric. This asymmetry generates a resistance to motion that contributes to the friction force. This friction force and its dependence on indenter geometry have not been systematically quantified.

Another contribution to the total friction force in adhesive contacts arises from the contact area, due to the shear stress required to slide the interior of the contact [11]. Although this area contribution has been widely studied and is often described by a simple empirical law of constant shear stress inside the contact region, the present work focuses exclusively on the boundary-line contribution to friction discussed above.

In this study, we investigate how this boundary-driven friction depends on the geometry of the indenter in tangential contact of viscoelastic materials. The indenter is described by a power-law profile, which allows the analysis of different geometries including parabolic, conical, and sharp-tip. Our previous experimental work [15] also reported the influence of geometry on friction but considered mainly area contribution, and the current study provides the necessary theoretical framework and numerical validation to isolate the boundary-driven adhesion mechanism and its corresponding scaling laws.

Numerical simulations are conducted using the Fast Fourier Transformation (FFT)-assisted Boundary Element Method (BEM). Over the past few decades, the BEM has established itself as a robust and widely used tool in contact mechanics [16,17]. In particular, the development of the FFT-assisted BEM has provided a highly efficient framework for capturing complex interface behaviors, such as rough surface contacts and partial sliding in elastic or viscoelastic materials, as well as in functionally graded materials and coatings [18,19,20]. Furthermore, this method has been extensively developed for adhesive contacts, where surface interactions are modeled using the JKR theory, Maugis–Dugdale model, or Lennard-Jones potential [21,22,23,24]. Building on these advancements, we employ numerical simulations based on the FFT-assisted BEM specifically tailored for JKR-type adhesive contact [24] to analyze the quasi-static sliding contact between a rigid indenter and a viscoelastic half-space. Our results demonstrate that the friction force is strongly coupled with the indenter geometry, following distinct scaling relations with the normal load depending on the power-law index.

## 2. Method

**Figure 1:**
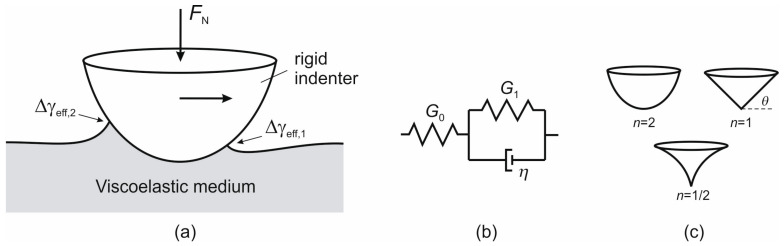
(**a**) Schematic of adhesive sliding contact between a rigid indenter and a viscoelastic half-space. (**b**) Rheological model of a linear viscoelastic material: the standard linear solid. (**c**) Profiles of three power-law indenters: parabolic (*n* = 2), conical (*n* = 1) and sharp (*n* = 0.5) indenters.

We consider the adhesive contact between a rigid indenter and a viscoelastic half-space (Figure 1a). The indenter geometry is described by a power-law profile *z* = *c* · *r^n^*. For *n* = 2, the indenter has a parabolic shape; for *n* = 1, it corresponds to a conical indenter; and for *n* = 1/2, it represents a sharp indenter tip, as shown in Figure 1c. The rheological behavior of the viscoelastic medium can be described by an arbitrary linear viscoelastic model, such as a generalized Prony series or the standard linear model. In this study, the standard model is adopted to illustrate the fundamental mechanisms, as shown in Figure 1b. Here, *G*_0_ is the long-term shear modulus, while *G*_1_ and *η* denote the shear modulus and viscosity of the Kelvin element, respectively.

The indenter is first pressed into the viscoelastic medium and then moves tangentially along the surface. Adhesion is taken into account in contact and is characterized by the material parameter Δγ, which represents the specific work of adhesion and is assumed to be independent of the loading parameters.

In [24], a stress criterion for determining the detachment and attachment of contact elements was proposed for the simulation of JKR-type adhesive contacts of elastic materials within the framework of the Boundary Element Method (BEM):

(1) $$\sigma_{c}=-\sqrt{\frac{E^{*}\Delta \gamma }{0.473201\cdot \Delta }},$$

where E* is the effective elastic modulus of the half-space and Δ is the size of the square discrete elements. During the simulation, if the tensile stress at the contact boundary exceeds this critical stress, the corresponding element detaches from the contact. The numerical scheme adopted here has been successfully applied and validated in our previous study [24]. This method can reproduce the classical JKR solution for elastic adhesive contact of a smooth parabolic indenter and has been widely used in simulations of rough surface contact [11,25].

We now consider *quasi-static viscoelastic* contact. The method described above for elastic contacts can be directly extended to the simulation of viscoelastic contacts. In a recent study, an energetic criterion was proposed for quasi-static adhesive normal contact of viscoelastic bodies. For the linear viscoelastic model, the elastic modulus and the work of adhesion in Equation (1) are replaced by the following expressions:

(2) $$E^{*}\to 4\frac{G_{0}G_{1}}{G_{0}+G_{1}},$$

(3) $$\Delta \gamma \to \Delta \gamma_{eff,1}=\Delta \gamma \frac{G_{1}}{G_{0}+G_{1}}\;\mathrm{for}\;\mathrm{attachment}\;(\mathrm{indentation}),$$

(4) $$\Delta \gamma \to \Delta \gamma_{eff,2}=\Delta \gamma \frac{G_{0}+G_{1}}{G_{1}}\;\mathrm{for}\;\mathrm{detachment}.$$

It should be noted that the elastic modulus E^*^ is identical in both the indentation (attachment) and pull-off (detachment) phases, whereas the values of the effective work of adhesion Δγ_eff,1_ and Δγ_eff,2_ differ significantly. The factor “4” in Equation (2) arises from the standard relationship between the effective contact modulus E* and the shear modulus for incompressible materials with the Poisson’s ratio *ν* = 0.5. Since the shear modulus *G*_0_ is typically much larger than *G*_1_, the factor *G*_1_/(*G*_0_ + *G*_1_) in Equation (3) produces a weak adhesion effect during the indentation phase, while (*G*_0_ + *G*_1_)/*G*_1_ in Equation (4) results in a very strong adhesion effect during pull-off. Consequently, pronounced adhesion hysteresis can be observed. The energetic criterion assumes that the short-range nature of adhesion allows for a clear separation between the fast elastic detachment and slow viscous relaxation. While this is typically satisfied for the trailing edge (detaching) at standard sliding velocities, a more detailed consideration of the material’s relaxation spectra may be necessary for the leading edge (indenting). The limitations of the applicability of this energy criterion were discussed in [13].

This behavior has been observed in numerous experiments [26,27], where indentation is close to Hertzian contact without adhesion effect. The difference between these effective works of adhesion gives rise to adhesion hysteresis and friction acting along the contact boundary.

In normal contact, elements at the contact boundary come into contact during indentation, corresponding to “crack closing” in fracture mechanics, while they detach during pull-off, corresponding to “crack opening”.

The same concept can be applied to tangential contact. When the indenter slides over an elastic or viscoelastic medium, elements at the leading edge of the contact enter contact, corresponding to the indentation phase in normal contact, whereas elements at the trailing edge detach, corresponding to the pull-off phase. Consequently, the elements at the leading edge (attaching) are governed by the effective work of adhesion Δγ_eff,1_ (Equation (3)), whereas the elements at the trailing edge (detaching) are governed by the effective work of adhesion Δγ_eff,2_ (Equation (4)).

In normal contact, the elements at the contact boundary come into contact during the indentation, corresponding to “crack closing” in fracture mechanics, and the elements detach during pull-off, corresponding to “crack opening”. This approach is also valid in tangential contact: when the indenter slides on the elastic or viscoelastic medium, the elements in the front of contact come into contact corresponding to the indentation phase in normal contact, and the elements at the rear detach which corresponds to pull-off phase. The frictional force arises from the difference between these two effective works of adhesion. According to the analysis in [11], the tangential force *F*_R_ is given by

(5) $$F_{R}=\left(\Delta \gamma_{eff,2}-\Delta \gamma_{eff,1}\right)\cdot D.$$

**Figure 2:**
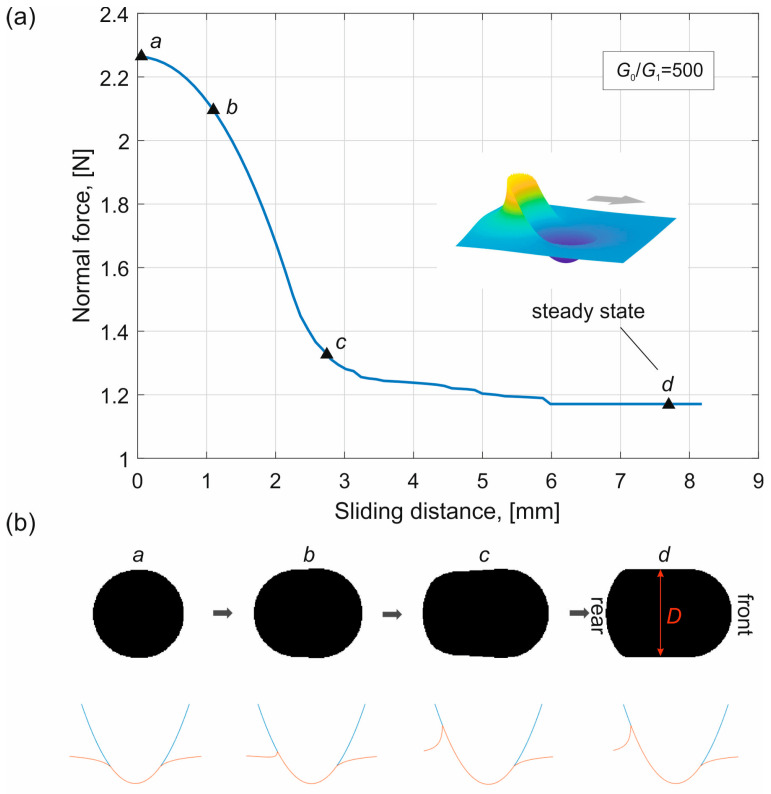
A simulation example of quasi-static adhesive tangential contact between a parabolic indenter and a viscoelastic half-space under indentation-depth-controlled conditions. (**a**) Evolution of the normal force during sliding. (**b**) Contact area and the cross-sections of contact configuration during sliding at four selected positions. The simulation parameters are *G*_0_/*G*_1_ = 500 and indentation depth *d* = 0.033 mm.

The parameter D, hereafter referred to as the contact size, is defined as the maximum width of the contact area in the direction perpendicular to the sliding direction (see the schematic illustration in Figure 2b). The two values of effective work of adhesion are given in Equations (3) and (4). Consequently, the contact size *D* must be determined numerically.

## 3. Analytical Estimation

Before performing the numerical simulations, we provide a brief estimate of the tangential force for indenters sliding on a viscoelastic medium. As discussed in the previous section, the effective work of adhesion Δγ_eff,1_ is very weak for the elements at the leading edge of the contact region, which are about to come into contact. Therefore, contact in the front region can be approximated as a simple Hertzian contact.

We assume that the size of the contact area in Equation (5) is primarily determined by the indentation phase, which will be confirmed later in the simulations. When an indenter with a power-law profile *z* = *c* · *r^n^* is pressed into an elastic half-space under the normal load *F*_N_, the relationship between the contact force and contact radius is given by [1,28]

(6) $$F_{N}=E^{*}\frac{2n}{n+1}\cdot \kappa \left(n\right)\cdot c\cdot a^{n+1},$$

where *a* is contact radius, $\kappa \left(n\right)=\sqrt{\pi }\frac{\Gamma \left(n/2+1\right)}{\Gamma \left(\left(n+1\right)/2\right)}$, and $\Gamma \left(.\right)$ is gamma function.

For quasi-static viscoelastic contact, substituting the effective elastic modulus from Equation (2) into Equation (6) yields

(7) $$F_{N}=4\frac{G_{0}G_{1}}{G_{0}+G_{1}}\frac{2n}{n+1}\cdot \kappa \left(n\right)\cdot c\cdot a^{n+1}.$$

Substituting the contact size *D* = 2*a* from Equation (7) into Equation (5), we obtain the dependence of the frictional force on the normal load

(8) $$F_{R}=2\cdot \left(\Delta \gamma_{eff,2}-\Delta \gamma_{eff,1}\right)\cdot {\left(\frac{G_{0}+G_{1}}{8G_{0}G_{1}}\cdot \frac{n+1}{n}\cdot \frac{1}{c\cdot \kappa \left(n\right)}\cdot F_{N}\right)}^{1/\left(n+1\right)}.$$

For the three indenters shown in Figure 1c, the corresponding frictional force is listed below:

(1) Parabolic indenter (*n* = 2) with sphere radius *R*, $\kappa \left(2\right)=2$, *c* = 1/(2*R*),
  (9) $$F_{R}=\left(\Delta \gamma_{eff,2}-\Delta \gamma_{eff,1}\right)\cdot {\left(\frac{3}{2}\frac{G_{0}+G_{1}}{G_{0}G_{1}}RF_{N}\right)}^{1/3}.$$
(2) Conical indenter (*n* = 1) with angle *θ* (definition of angle *θ* is shown in Figure 1c), $\kappa \left(1\right)=\pi /2$, *c* = tan*θ*,
  (10) $$F_{R}=\left(\Delta \gamma_{eff,2}-\Delta \gamma_{eff,1}\right)\cdot {\left(\frac{2}{\pi }\frac{G_{0}+G_{1}}{G_{0}G_{1}}\frac{F_{N}}{\tan\theta }\right)}^{1/2}.$$
(3) Sharp-tip indenter (*n* = 1/2) with constant *c*, $\kappa \left(1/2\right)\approx 1.311$,
  (11) $$F_{R}\approx 2.188\cdot \left(\Delta \gamma_{eff,2}-\Delta \gamma_{eff,1}\right)\cdot {\left(\frac{G_{0}+G_{1}}{4G_{0}G_{1}}\frac{F_{N}}{c}\right)}^{2/3}.$$

## 4. Numerical Results

We numerically simulate quasi-static tangential contact for these three indenters. In all simulations, the shear modulus *G*_1_ = 10 MPa and the specific work of adhesion Δγ = 5 × 10^−5^ N/mm are kept constant. The ratio of *G*_0_/*G*_1_ is varied from 1 to 1000, which leads to different values of the effective work of adhesion Δγ_eff,1_ and Δγ_eff,2_, as listed in Table 1. It can be seen that a large ratio of *G*_0_/*G*_1_ results in a significant difference between the effective works of adhesion at the leading and trailing edges.

### 4.1. Parabolic Indenter (n = 2)

In the case of a parabolic indenter, the sphere radius is set to *R* = 50 mm, corresponding to a profile factor *c* = 1/(2*R*). During the indentation phase, the effective work of adhesion Δγ_eff,1_ is used (elements attach only). During sliding, Δγ_eff,1_ and Δγ_eff,2_ are applied separately to elements that attach and detach, respectively.

Figure 2 shows a simulation example of the tangential sliding phase under indentation-depth-controlled conditions for *G*_0_/*G*_1_ = 500. The first image in Figure 2b shows the contact configuration before sliding (i.e., immediately after indentation). The contact area still has a circular shape. From the cross-section of the contact configuration shown below, it can be seen that almost no adhesion effect occurs at the contact boundary, and the contact is close to Hertzian due to the small value of Δγ_eff,1_.

During sliding, elements at the leading edge of the contact enter contact, and the contact region in the front maintains approximately the same shape. However, at the trailing edge, due to the larger effective work of adhesion Δγ_eff,2_, the elements remain adhered to the indenter, causing the contact region to become “stretched” (see Figure 2b, point c). From the cross-section of the contact configuration, the adhesion effect at the trailing edge can be clearly observed, which leads to a reduction in the normal force during sliding.

The final steady state is shown in Figure 2b, point d. The surface displacement of the viscoelastic half-space is illustrated in Figure 2a, where the arrow indicates the sliding direction. In the following, we focus on the relationship between the normal force *F*_N_ and the contact size *D* in the final steady state. It is noted that *D* is the size of contact perpendicular to sliding direction as shown in image (d) in Figure 2b.

**Figure 3:**
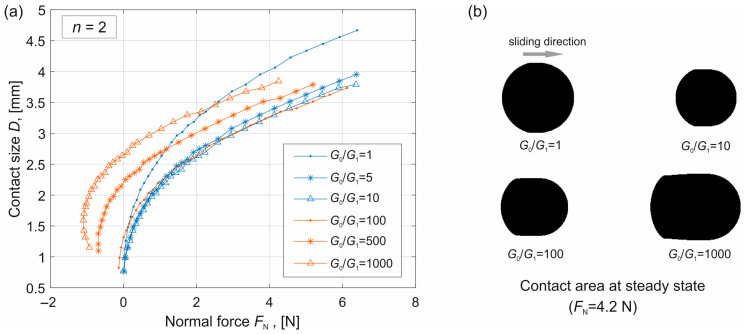
(**a**) Dependence of the contact size *D* on the normal force at the steady sliding state for different values of *G*_0_/*G*_1_. (**b**) Contact area in the case of four different values of *G*_0_/*G*_1_ but with the same normal force.

The simulations were carried out for different indentation depths and different values of *G*_0_/*G*_1_. The dependence of the contact size on the normal force in the steady state is shown in Figure 3a. It can be seen that for small values of *G*_0_/*G*_1_, (blue curves, corresponding to *G*_0_/*G*_1_ = 1, 5 and 10), the normal force remains almost always positive (compressive force). In this range, the contact size decreases as *G*_0_ increases.

However, for larger values of *G*_0_/*G*_1_ (red curves), the normal force can also become negative (tensile force), and the contact size increases with increasing normal force. This phenomenon is attributed to the pronounced adhesion at the trailing edge of the contact, which is governed by the high effective work of adhesion Δγ_eff,2_ at these large ratios. This behavior can also be observed in Figure 3b, where the contact area at the same normal load but for different values of *G*_0_/*G*_1_ is shown.

The reason is that when *G*_0_/*G*_1_ is small, the adhesion effect is weak, and the contact size is mainly governed by Hertzian contact. In this case, the effective elastic modulus E* = 4*G*_0_*G*_1_/(*G*_0_ + *G*_1_) increases with increasing *G*_0_, and therefore the contact size predicted by Hertzian contact theory becomes smaller. However, when *G*_0_ is large, adhesion at the trailing edge plays a more significant role, leading to a larger contact area.

**Figure 4:**
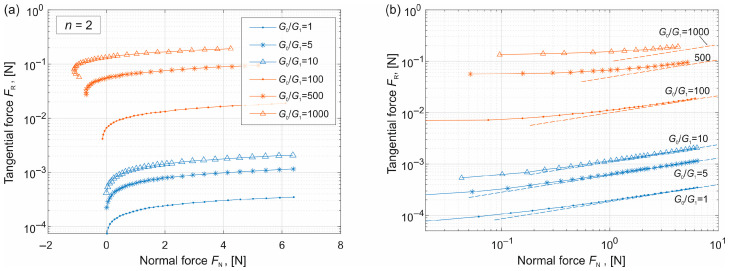
(**a**) Dependence of the tangential force on the normal force for different values of *G*_0_/*G*_1_. (**b**) Comparison of numerical results with analytical estimation given in Equation (9) (dashed lines).

Using the obtained contact size, the tangential force *F*_R_ is calculated according to Equation (5). The dependence of the tangential force on the normal force is shown in Figure 4a. To clearly present the tangential force, the y-axis is plotted on a logarithmic scale. It can be seen that the tangential force increases with increasing normal force, and it becomes larger for higher values of *G*_0_/*G*_1_.

To compare the results with the analytical estimation given in Equation (9), the relationship is replotted in a double-logarithmic scale, where only positive values of the normal force are shown. The analytical solutions are shown with dashed lines. It can be observed that when the normal force is not small, the analytical solution provides a very good approximation, with *F*_R_ ∝ *F*_N_^1/3^. For large values of *G*_0_, however, the deviation from the analytical prediction becomes more significant.

### 4.2. Conical Indenter (n = 1) and Sharp-Tip Indenter (n = 1/2)

**Figure 5:**
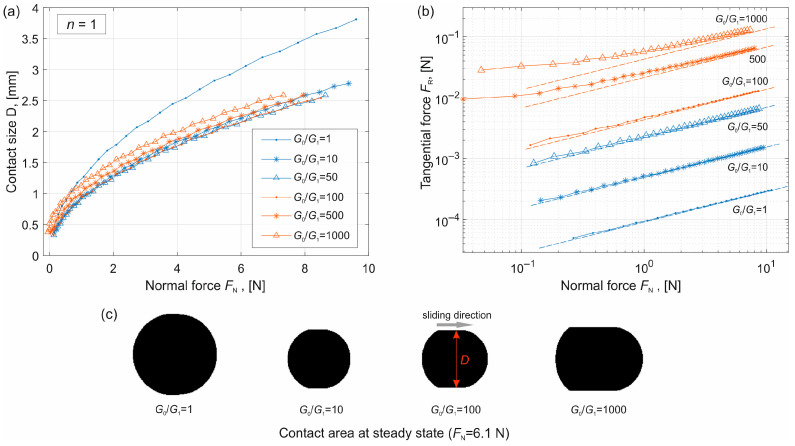
Results for conical indenter (*n* = 1). (**a**) Dependence of the contact size on the normal force for different values of *G*_0_/*G*_1_. (**b**) Dependence of the tangential force on the normal force. The dashed lines are the analytical estimation given in Equation (10). (**c**) Contact area at the steady state for different values of *G*_0_/*G*_1_ but with the same normal force *F*_N_.

**Figure 6:**
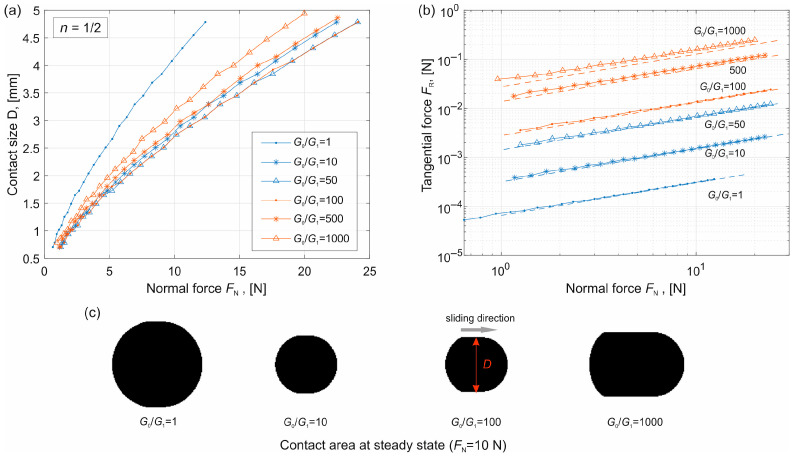
Results for sharp-tip indenter (*n* = 1/2). (**a**) Dependence of the contact size on the normal force for different values of *G*_0_/*G*_1_. (**b**) Dependence of the tangential force on the normal force. The dashed lines are the analytical estimation given in Equation (10). (**c**) Contact area at the steady state for different values of *G*_0_/*G*_1_ but with the same normal force *F*_N_.

Simulations were then carried out for a conical indenter (*n* = 1) with an angle *θ* = 5° (the definition of the angle is shown in Figure 1c), corresponding to a profile factor *c* = tan(*θ*). For a sharp-tip indenter (*n* = 1/2), the profile factor *c* = 0.2 mm^0.5^. The results are presented in Figure 5 and Figure 6 respectively.

In these figures, subplot (a) shows the dependence of the contact size on the normal force, subplot (b) shows the tangential force calculated according to Equation (5), and subplot (c) shows the contact area at the steady state for different values of *G*_0_/*G*_1_ but under the same normal force.

The results exhibit the same trends as in the case of the parabolic indenter. In particular, the contact size *D* first increases with increasing *G*_0_ (blue curves) and then decreases (red curves). Comparison with the analytical solutions given in Equations (10) and (11) (dashed lines) shows good agreement for sufficiently large normal loads.

It is noted that for the conical indenter (*n* = 1), *F*_R_ ∝ *F*_N_^1/2^, while for the sharp indenter (*n* = 1/2), *F*_R_ ∝ *F*_N_^2/3^.

## 5. Discussion and Conclusions

The present study investigated quasi-static adhesive tangential contact between a rigid indenter with a power-law profile and a viscoelastic half-space using the Boundary Element Method. Adhesion was incorporated through a stress-based detachment criterion with effective works of adhesion derived from an energetic approach. For linear viscoelastic material, the work of adhesion for detaching is much larger than that for indenting, which results in a Hertzian-like contact at the leading edge but strong adhesion at the trailing edge.

Based on these Hertzian-like contact conditions, analytical estimations for the friction force were derived. These theoretical models predict distinct scaling relations between the tangential and normal forces depending on the indenter geometry:

- *F*_R_ ∝ *F*_N_^1/3^ for parabolic indenter (*n* = 2);
- *F*_R_ ∝ *F*_N_^1/2^ for conical indenter (*n* = 1);
- *F*_R_ ∝ *F*_N_^2/3^ for sharp indenter (*n* = 1/2).

Numerical simulations conducted using the BEM reveal that the contact area becomes asymmetric during sliding and that the contact size and normal force depend strongly on the ratio *G*_0_/*G*_1_. The relations between friction and normal force for these three different indenters show excellent agreement with these analytical predictions, particularly in the regime of sufficiently large normal loads.

These results indicate the role of indenter geometry in adhesive friction of viscoelastic contacts and should be carefully considered when analyzing or designing tribological systems involving soft viscoelastic materials such as high-performance elastomeric seals, biomedical hydrogels, and tire tread compounds.

Finally, we emphasize that this study focused exclusively on the “boundary-driven contribution” to friction. While factors such as surface roughness, loading rate, and bulk viscoelastic dissipation play significant roles in real-world applications, their detailed analysis is beyond the scope of the present paper. By isolating the boundary hysteresis in a clean, smooth-surface limit, this work establishes a fundamental theoretical benchmark. This benchmark provides a necessary reference point for future research to systematically evaluate how more complex interfacial factors might modulate the underlying scaling laws derived here.

## Figures and Tables

**Table 1:** Ratio of *G*_0_/*G*_1_ and corresponding effective work of adhesion.

*G*_0_/*G*_1_	Δγ_eff,1_/Δγ	Δγ_eff,2_/Δγ
1	0.5000	2
10	0.0909	11
100	0.0099	101
1000	0.0010	1001

## Data Availability

The original contributions presented in this study are included in the article. Further inquiries can be directed to the corresponding authors.

## Abbreviations

The following abbreviations are used in this manuscript:

BEM	Boundary Element Method
